# COGNITIVE FUNCTION CONTRIBUTES TO THE NONLINEAR RELATIONSHIP BETWEEN AGE AND DUAL-TASK GAIT IN MID-AGED POPULATION

**DOI:** 10.1093/geroni/igac059.914

**Published:** 2022-12-20

**Authors:** Junhong Zhou, Gabriele Cattaneo, Wanting Yu, Natalia Gouskova, Lewis Lipsitz, Alvaro Pascual-Leone, David Bartres-Faz, Brad Manor

**Affiliations:** Harvard Medical School/Hebrew SeniorLife, Roslindale, Massachusetts, United States; Universitat Autònoma de Barcelona, Barcelona, Catalonia, Spain; Hebrew SeniorLife, Roslindale, Massachusetts, United States; Hebrew SeniorLife, Roslindale, Massachusetts, United States; Hebrew SeniorLife, Brookline, Massachusetts, United States; Hebrew SeniorLife, Roslindale, Massachusetts, United States; Institut Universitari de Neurorehabilitació adscrit a la UAB, Barcelona, Catalonia, Spain; Hebrew SeniorLife/Harvard Medical School, Roslindale, Massachusetts, United States

## Abstract

The capacity to maintain safe walking is critical to functional independence in older adults. However, the timing/stage when such capacity starts to diminish, and its potential contributors have not been well characterized. To explore that, we here conducted analysis based upon the data of 651 participants of age between 40 and 65 years from Barcelona Brain Health Initiative Study. Each participants completed: 1) one 45-second trial of walking normally (single-task) and while performing a serial-subtraction-by-three task (dual-task), of which gait was measured using a smartphone-based gait-assessment application; and 2) a battery of cognitive tests. The dual-task cost (DTC) (i.e., percent changes from single- to dual-task condition) to mean stride time (ST) and stride time variability (STV) and the score of global cognitive function were obtained. The LOESS analyses demonstrated nonlinear relationships between age and DTCs with a turning point at age of 54 years (R2>3%). Regression models showed significantly greater associations (p=0.01~0.03) between age and DTCs (i.e., older age, worse gait) (β=0.22~0.28, p< 0.006), as well as between global cognitive function and DTCs (β=-0.28~-0.18, p< 0.002), in older group (i.e., age≥54 years) compared to younger group. The structural-equation-modeling suggested that in older group, cognitive function mediated the relationship between age and dual-task gait (p< 0.02) with a contribution of 43~47% to such relationship. The observations here revealed that as early at age of 54 years, dual-task gait starts to significantly diminish, and its dependence on cognitive function dramatically increases, providing critical knowledge for the management of mobility and cognitive aging in mid-age population.

